# Amino acid regulation of peroxidase-like activity of Cu_2_O nanozyme for detection of tetracycline antibiotics

**DOI:** 10.1007/s00216-025-05904-1

**Published:** 2025-05-14

**Authors:** Yueqiang Wang, Shengwei Sun

**Affiliations:** 1 Faculty of Science, National University of Singapore, Block S16 Level 9, 6 Science Drive 2, Singapore, 117546 Singapore; 2 School of Engineering Sciences in Chemistry, Biotechnology and Health, Department of Fibre and Polymer Technology, KTH Royal Institute of Technology, 100 44 Stockholm, Sweden

**Keywords:** Nanozyme, Tetracycline antibiotics, Cu_2_O, Amino acids

## Abstract

Tetracycline antibiotics (TCs) are widely used in medicine, agriculture, and animal husbandry. However, their overuse has led to environmental pollution, posing a significant threat to water sources, soil, and food safety. Therefore, there is an urgent need for efficient, sensitive, simple, and low-cost detection methods for environmental pollution monitoring. In this study, the catalytic activity of copper-based nanozymes was regulated by AAs. Lysine, aspartic acid, glycine, and arginine were chosen as ligands to synthesize different copper-based nanozymes. The results showed that the type of amino acid significantly influenced the particle size, morphology, and peroxidase (POD)-like catalytic activity of Cu₂O. Based on these amino acid-regulated Cu₂O nanozymes, we further developed a highly sensitive, easy-to-use, and low-cost colorimetric sensor array that can effectively distinguish TCs. This sensor array was successfully validated in binary mixtures and wastewater environments. This study not only provides important insights into the small-molecule regulation of copper-based nanozyme catalytic performance but also offers a novel approach for the detection of TCs in environmental monitoring.

**Supplementary Information:**

The online version contains supplementary material available at 10.1007/s00216-025-05904-1.

## Introduction

Enzymes are renowned for their high selectivity, sensitivity, environmental friendliness, rapid response, and biocompatibility, making them ideal for catalyzing specific reactions under mild conditions amplifying detection signals, and identifying trace target molecules [1]. However, despite their high catalytic efficiency and specificity, their widespread application is limited by several drawbacks [2], including poor stability [3], susceptibility to environmental conditions [4], high production costs [5], single catalytic functions [6], and limited tunability [7]. In contrast, nanozymes offer significant advantages over natural enzymes, such as enhanced stability, which allows them to maintain activity under extreme temperatures, pH values, and organic solvents, as well as a longer shelf life [8]. They are also more cost-effective due to simpler and more economical production processes [9], and they possess versatile catalytic functions that can be tailored and optimized to meet diverse reaction requirements [10]. Currently, the catalytic centers of nanozymes mainly consist of metals with variable oxidation states, such as iron, gold, platinum, manganese, silver, and cobalt [11, 12]. Despite these advantages, regulating the catalytic activity of nanozymes remains a significant challenge [13].

The activity of nanozymes can be regulated through various strategies, including surface modification, optimization of synthesis conditions [14] (such as adjusting temperature, pH, and precursor concentration), doping [15], and surface charge regulation [16]. For instance, Li et al. prepared nitrogen-doped Mn₃O₄ using different amino acids (AAs) as nitrogen sources, which exhibited oxidase-like activity. Further studies revealed that the N-doped Mn₃O₄ synthesized using histidine as the precursor demonstrated the highest oxidase-like activity [17]. Despite the availability of several methods to enhance nanozyme activity, most of them involve complex synthesis steps [18–20]. Therefore, developing simplified methods for the rapid synthesis of nanozymes with controllable activity is of significant importance.

Tetracycline antibiotics (TCs), widely used in medicine, agriculture, and animal husbandry, have raised significant concerns due to their overuse and environmental persistence, leading to contamination of water, soil, and food sources [21]. This widespread residue poses risks to human health and ecological systems and contributes to the growing issue of antibiotic resistance [22]. The detection of tetracycline residues is crucial for monitoring environmental pollution, ensuring food safety, and preventing the spread of resistant bacteria [23]. However, current detection methods, such as high-performance liquid chromatography (HPLC) and mass spectrometry (MS), are often complex, time-consuming, and costly [24]. Therefore, there is a pressing need for the development of novel, rapid, and cost-effective solutions to address these challenges effectively.

**Scheme 1:**
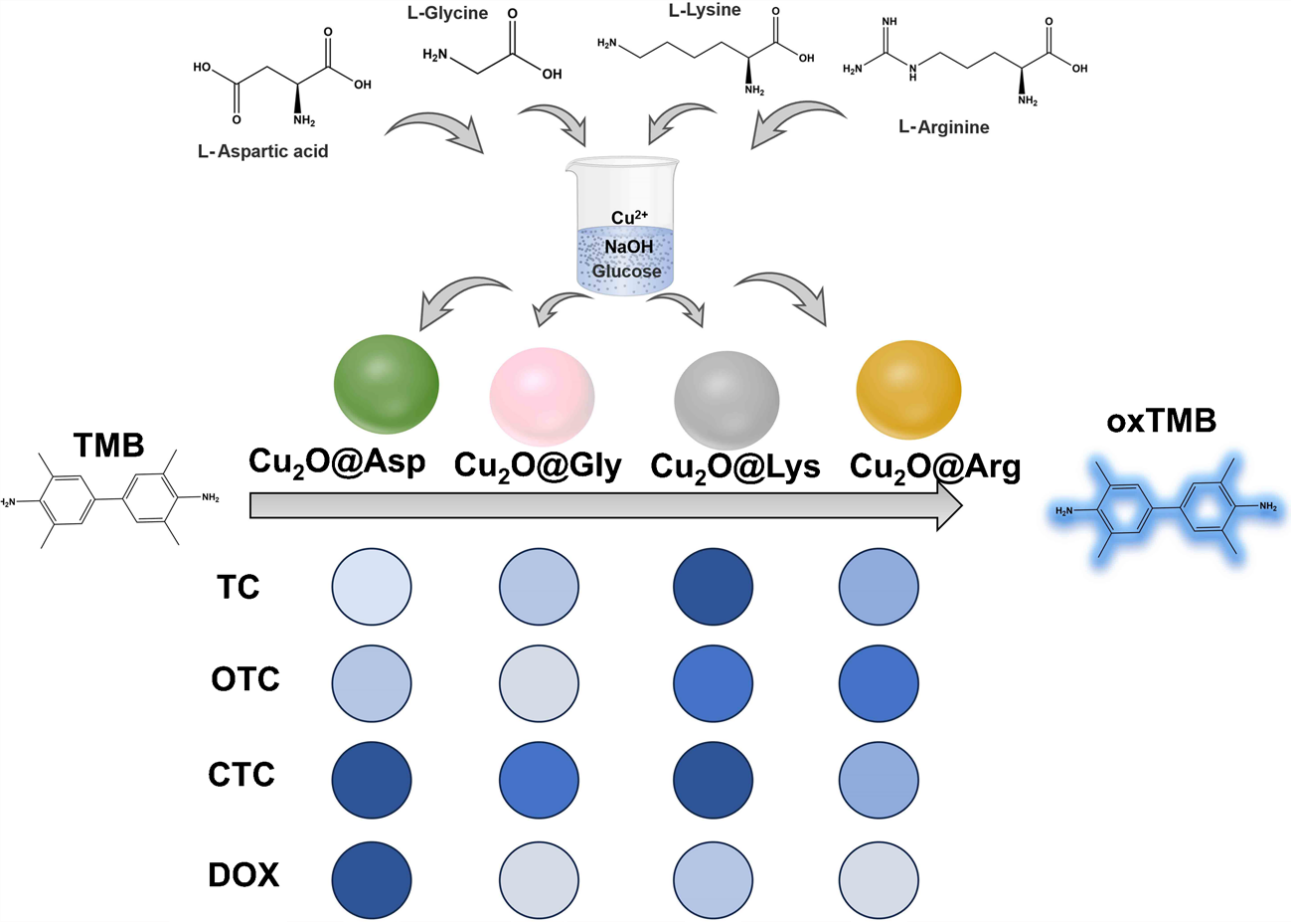
Schematic representation of different AA-modified Cu₂O@AA used to construct colorimetric sensing arrays for the detection of four TCs

This study investigates the regulatory effect of AA on the synthesis of copper(I) oxide (Cu₂O) and examines the impact of AA on the morphology and peroxidase(POD)-like catalytic activity of Cu₂O. By adjusting the type of four different AAs, we found that AA significantly influenced the particle size, morphology, and catalytic activity of Cu₂O, particularly in regulating its POD-like activity. This makes it show great potential applications in the field of biosensing and environmental monitoring [25]. Furthermore, we applied this AA-regulated Cu₂O system for the detection of TCs. Four commonly used TCs, such as tetracycline (TC), doxycycline (DOX), chlortetracycline (CTC), and oxytetracycline (OTC), exhibited different responses on the Cu₂O surface due to structural differences (Scheme 1). By exploiting these differential responses, we developed a colorimetric sensor array for distinguishing and detecting TCs. This sensor array allows for quantitative detection of antibiotic concentrations through color changes, with high sensitivity, simple operation, and low cost. This study demonstrates the potential of AA-regulated Cu₂O materials for antibiotic detection, offering new insights for developing efficient, sensitive, and cost-effective detection methods. These methods show broad applications, particularly in environmental monitoring and food safety.

## Experimental section

### Reagents and materials

Glycine (Gly), L-arginine (Arg), L-aspartic acid, L-lysine, tetracycline (TC), chlortetracycline (CTC), oxytetracycline (OTC), doxycycline (DOX,) and 3,3′,5,5′-tetramethylbenzidine (TMB) were purchased from Sigma-Aldrich (Sigma-Aldrich, Singapore). All chemicals and reagents used in the experiments were unpurified raw materials and used directly. The water used in the experiments was deionized (DI) water.

### Characterization

The crystal structure was analyzed using an XRD (Bruker D8 Venture) instrument. UV signals were recorded using a Shimadzu UV-2800 spectrophotometer (Shimadzu, Japan). Fourier transform infrared (FT-IR) spectra were obtained with a Varian 3100 FT-IR spectrometer (USA). XPS measurements were conducted on a PHI 5000 instrument. Scanning electron microscopy (SEM) images were taken using a FET Quanta 400 FEG (Hitachi SU-8220).

### Synthesis of Cu_2_O@Arg, Cu_2_O@Gly, Cu_2_O@Asp, and Cu_2_O@Lys

Dissolve 10 mM CuCl₂·2H₂O and 2 mM L-Arg, L-Lys, L-Asp, and L-Gly separately in 50 mL of deionized water to obtain a blue solution. Add 5 mM glucose to the solution, heat it to 80 °C, and slowly add 20 mL of 2 M NaOH dropwise. Stir the mixture in a water bath for 1 h, then centrifuge, and wash the product. Finally, disperse the product in deionized water. The yields of Cu_2_O modified by different AAs range from 50 to 90%.

### Determination of the POD-like activity

The POD-like activity of nanozyme was evaluated using the colorimetric reaction between TMB and H₂O₂ [26]. Briefly, 40 μL of Cu₂O@Arg, Cu₂O@Gly, Cu₂O@Asp, or Cu₂O@Lys (40 μg·mL⁻^1^), 10 μL of H₂O₂ (90 mM), 10 μL of TMB (0.2 mM), and 940 μL of sodium acetate-acetic acid (NaAc-HAc) buffer (10 mM, pH 5.0 or 6.0) were sequentially added. The NaAc-HAc buffer (10 mM, pH 5.0 or 6.0) was prepared by mixing appropriate amounts of sodium acetate and acetic acid, and the pH was adjusted with NaOH or HCl as needed. After thorough mixing, the reaction was incubated for 1 h. The UV–Vis absorbance at 650 nm was then measured, and the catalytic activity of the catalysts was determined based on the absorbance intensity.

The steady-state kinetics of Cu₂O@Arg, Cu₂O@Gly, Cu₂O@Asp, and Cu₂O@Lys were evaluated by monitoring absorbance at 650 nm (*ε* = 39,000 M⁻^1^·cm⁻^1^). Briefly, varying concentrations of H₂O₂ (5, 10, 20, 30, 50, 60, 80, and 90 mM) were mixed with Cu₂O@Arg, Cu₂O@Gly, Cu₂O@Asp, or Cu₂O@Lys (40 μg·mL⁻^1^) and TMB (0.2 mM), and the absorbance changes at 650 nm were recorded continuously for 60 s.

Subsequently, varying concentrations of TMB (0.005, 0.01, 0.02, 0.04, 0.05, 0.1, 0.15, and 0.2 mM) were mixed with Cu₂O@Arg, Cu₂O@Gly, Cu₂O@Asp, or Cu₂O@Lys (40 μg·mL⁻^1^) and H₂O₂ (90 mM), and the absorbance changes at 650 nm were again monitored continuously for 60 s. Kinetic parameters were determined by fitting the data to the Michaelis–Menten equation.

$${\mathrm{V}}\mathrm{ = }\frac{{\mathrm{V}}_{\mathrm{max}}[{\mathrm{S}}]}{{\mathrm{K}}_{\mathrm{m}}\mathrm{ + }\mathrm{[}{\mathrm{S}}\mathrm{]}}$$

*V* is the initial velocity; *V*_max_ is the maximal reaction velocity; [*S*] is the substrate concentration; *K*_m_ is the Michaelis constant.

### A colorimetric sensor array was constructed to detect TCs

A colorimetric sensor array was constructed for the detection of TCs (OTC, CTC, TC, and DOX) as analytes. First, four materials, Cu₂O@Arg, Cu₂O@Gly, Cu₂O@Asp, and Cu₂O@Lys (40 μg·mL⁻^1^), were mixed with varying concentrations of the analytes and incubated at room temperature for 30 min. Then, 930 μL of NaAc-HAc buffer (10 mM, pH 5.0) and TMB (0.2 mM) was added and thoroughly mixed. The mixture was further incubated for 20 min until the reaction was complete. The absorbance of the samples at 650 nm was measured using a UV–Vis spectrophotometer. Each experiment was repeated three times, resulting in a 4 × 4 × 3 data matrix (4 materials × 4 analytes × 3 replicates). The data obtained were then analyzed using the principal component analysis (PCA) function using Origin 2023 software (Student Edition) and the score plots obtained were used to determine the distribution of the substances.

### Real samples analysis

A sample of municipal wastewater from Singapore was collected and used as the substrate for real-sample analysis. Impurities were removed using a membrane with a pore size of 0.22 μm. CTC, OTC, DOX, and TC were spiked into the effluent samples at a concentration of 20 μM using the standard addition method, followed by analysis with the constructed colorimetric sensing array. The resulting data were processed and evaluated using PCA with the original student-version software.

## Results and discussion

### Structural characterization

**Fig. 1:**
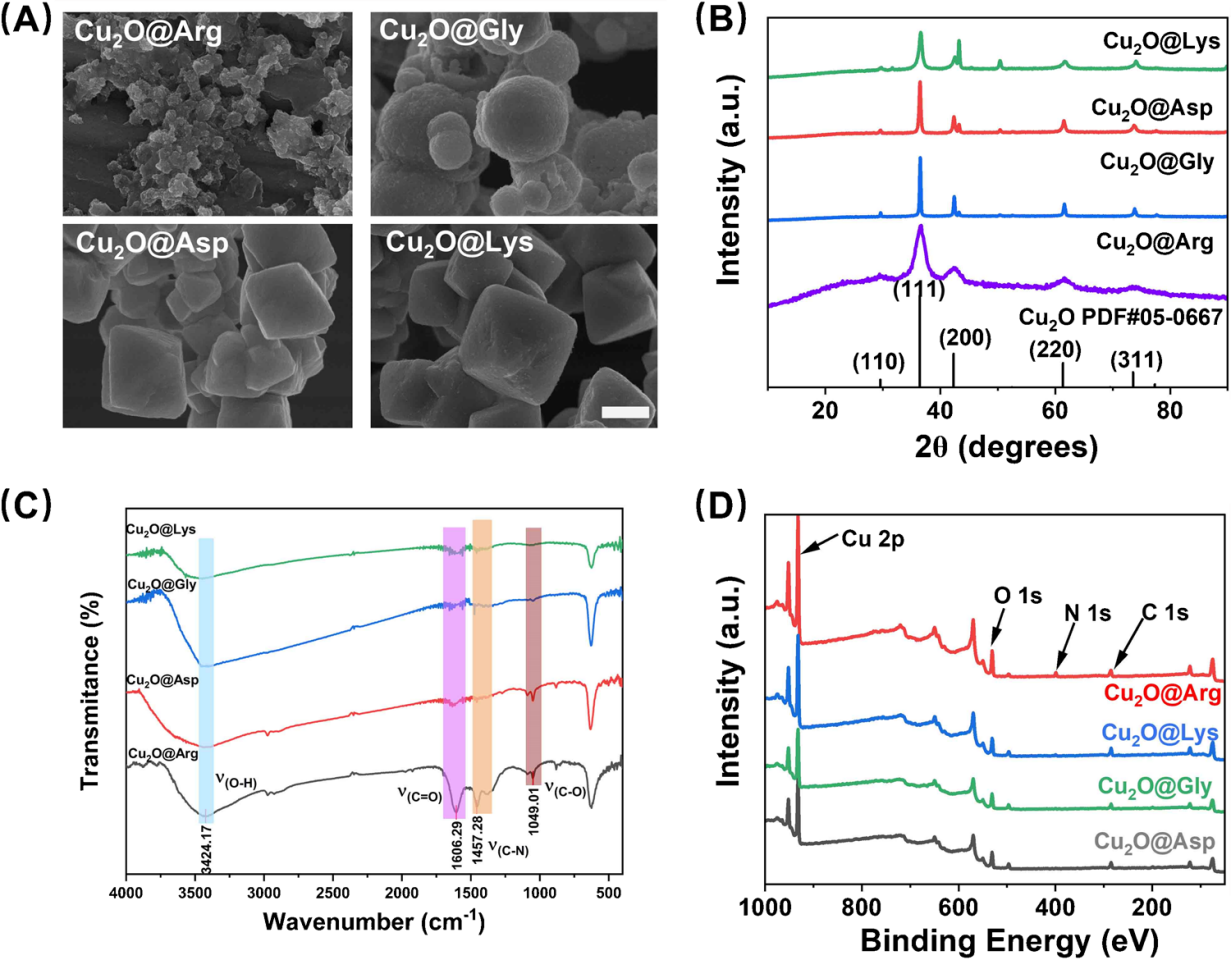
**A** SEM image (scale bar, 200 nm), **B** XRD spectrum (standard card for Cu_2_O is PDF#05–0667), **C** FTIR spectrum, and **D** XPS full spectrum of Cu₂O particles with different AAs modifications

We first synthesized Cu₂O regulated by different AA via a glucose reduction method [24]. The morphology, structure, and surface chemistry of Cu₂O were systematically analyzed using scanning electron microscopy (SEM), X-ray diffraction (XRD), Fourier-transform infrared spectroscopy (FTIR), and X-ray photoelectron spectroscopy (XPS). As shown in Fig. 1A, different AA significantly influenced the morphology and particle size of Cu₂O. L-Arg-synthesized Cu₂O presents a lamellar structure, suggesting that arginine may direct growth through strong interaction with specific crystal faces [24]. The Cu₂O synthesized by L-Gly was in the form of spherical with rough surface and the particle size was about 200 ~ 800 nm. Both L-Lys- and L-Asp acid-modulated Cu₂O exhibit an octahedral morphology with a smooth surface. Transmission electron micrographs show agreement with SEM results (Figure S1). In addition, the hydrodynamic particle size measurements closely matched the results obtained from electron microscopy (Figure S2). The significant modulation of Cu₂O crystal size and morphology by AA is primarily driven by the structure-directed effects of different AA molecules during the crystal growth process [27, 28]. Specifically, the unique molecular configurations—such as the spatial arrangement of carboxyl and amino groups, the ability to form hydrogen bonding networks, and the chiral properties of AAs—play key roles in influencing the crystallization of Cu₂O.

XRD patterns (Fig. 1B) confirmed the cubic crystalline structure of Cu₂O, with characteristic diffraction peaks at 2*θ* = 29.6°, 36.4°, and 42.3°, corresponding to the 110, 111, and 200 crystal planes. Subtle differences in peak broadening and intensity were observed among the samples. For instance, Cu₂O synthesized with arginine exhibited significantly broadened peaks, indicating lower crystallinity and higher surface defect densities, consistent with the sheet-like structures observed in SEM. FTIR spectra (Fig. 1C) revealed characteristic absorption peaks associated with AA interactions on the Cu₂O surface. Peaks at 1606.29 cm⁻^1^, 1457.28 cm⁻^1^, and 1049.01 cm⁻^1^ were attributed to the stretching vibrations of C = O, C-N, and C-O groups, respectively, indicating the presence of AA on the surface. Notably, strong absorption in the range of 3200–3500 cm⁻^1^ was attributed to the stretching vibrations of carboxyl groups [29], further confirming the adsorption of AA. Furthermore, thermogravimetric analysis (TGA) revealed that among the tested AAs, L-arginine exhibited the highest adsorption onto Cu₂O under identical loading conditions (Figure S3). Consistently, Brunauer–Emmett–Teller (BET) measurements demonstrated that Cu₂O@L-Arg possessed a markedly increased specific surface area, whereas Cu₂O modified with other AAs displayed minimal or undetectable surface areas (Figure S4). XPS survey spectra (Fig. 1D) showed the elemental composition and chemical states of Cu₂O [30]. Peaks corresponding to Cu, O, N, and C were observed in all samples, further verifying the successful modification of Cu₂O by AA.

**Fig. 2:**
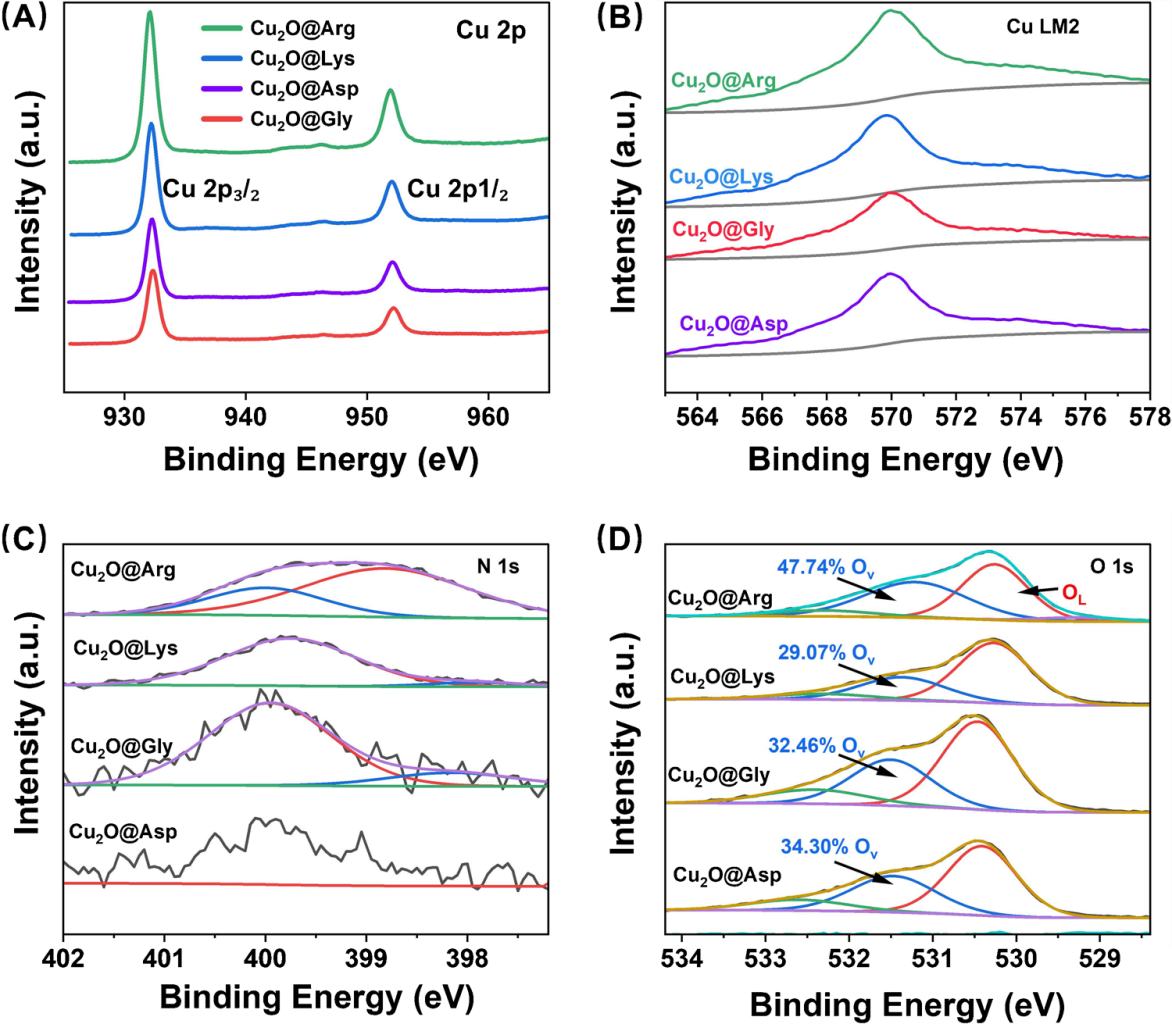
Cu_2_O@Arg, Cu_2_O@Lys, Cu_2_O@Gly, and Cu_2_O@Asp in **A** Cu 2p fine, **B** Cu LM2, **C** N 1 s fine, and **D** O 1 s fine spectra

Through XPS analysis of Cu₂O modified with different AA (Arg, Lys, Gly, andAsp), detailed insights into its surface elemental composition and chemical states were obtained [31]. The Cu 2p spectra exhibited characteristic peaks corresponding to Cu(I) species (Fig. 2A). All samples showed Cu 2p₃/₂ peaks around 932.5 eV, confirming the presence of Cu₂O, with no significant signals indicative of Cu(II) oxidation. Additionally, the absence of distinct satellite peaks further corroborated this conclusion. The slight variations in peak intensity among the samples suggested that the surface copper composition or coordination was influenced by the type of AA used for modification. The Auger Cu LM2 spectra, ranging from 566 to 576 eV, further verified the oxidation state of copper (Fig. 2B). All samples demonstrated that Cu(I) was the dominant species, and the slight shifts in binding energy indicated that the AA affected the local chemical environment of copper.

The N 1s spectra revealed the incorporation of nitrogen-containing groups from AA onto the surface of Cu₂O (Fig. 2C). For Cu₂O@Arg, a distinct peak at approximately 399.5 eV corresponded to -NH or -NH₂ groups. Additionally, the N 1 s spectrum could be fitted into two characteristic peaks, indicating the formation of abundant nitrogen-rich environments from L-Arg on the Cu₂O surface [15]. Similar -NH or -NH₂ peaks were observed for the products modified with lysine, glycine, and aspartic acid, although the intensities varied, reflecting differences among the modifiers.

The O 1s spectrum reveals the types of oxygen species present on the Cu₂O surface [32]. The two dominant peaks correspond to oxygen vacancies (O_v_) (~ 530 eV) and lattice oxygen (O_L_) (~ 531.5 eV) (Fig. 2D). The relative proportions of *O* v and *O* L varied across different samples, with Cu₂O@Arg exhibiting the highest proportion of oxygen vacancies (47.74%) and lower proportions for Cu₂O@Lys (29.07%), Cu₂O@Gly (32.46%), and Cu₂O@Asp (34.30%). This suggests that L-Arg modification induces a higher proportion of oxygen vacancies, potentially leading to lattice distortion, a phenomenon consistent with the XRD analysis. In summary, XPS analysis demonstrated that AA modifications significantly altered the chemical environment and surface composition of Cu₂O. Notably, changes in nitrogen and oxygen species align with the observed modulation of Cu_2_O morphology and catalytic activity.

### Catalytic activity of Cu_2_O@AA

**Fig. 3:**
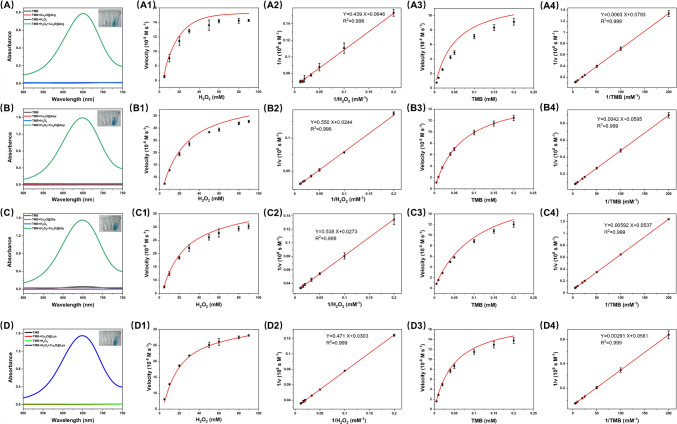
**A**-**D** UV–visible absorption spectra catalyzed by different substrates. Steady-state kinetic assay of the Cu_2_O@AA nanozyme for H_2_O_2_ (A1–D1) and TMB (A3–D3). POD-like kinetics data fitted using the Lineweaver–Burk model for H_2_O_2_ (A2–D2) and TMB (A4–D4)

The impact of different AA modifications on the enzyme-like activity of Cu₂O was further evaluated. UV–visible spectra showed that when TMB was present alone, its absorbance was almost zero (Fig. 3A-D), indicating that TMB cannot undergo oxidation in the absence of a catalyst or H₂O₂. When only nanozymes or H₂O₂ were added, the absorbance remained low, suggesting that neither of these components alone was sufficient to oxidize TMB. However, when both nanozymes and H₂O₂ were added together, the absorbance significantly increased, peaking around 650 nm, and the solution turned visibly blue. This phenomenon indicates that Cu₂O modified with different AA can effectively catalyze the oxidation of TMB in the presence of H₂O₂, exhibiting notable POD-like activity. By comparing the spectral intensities of four modified nanozymes and the unmodified Cu₂O at 650 nm under the same reaction conditions, we found that the catalytic activity improvement varied among Cu₂O@Arg, Cu₂O@Gly, Cu₂O@Asp, and Cu₂O@Lys. Among them, Cu₂O@Asp exhibited the highest catalytic activity (Figure S5).

To further explore the effect of AA modifications on nanozyme performance, the study systematically assessed the influence of pH and temperature on the catalytic activity through condition optimization experiments. The effect of pH on enzyme activity (Figure S6) revealed that the relative activity of the different modified nanozymes exhibited a typical “bell-shaped curve” within the pH range of 3 to 7. Notably, all modified Cu₂O displayed optimal catalytic activity between pH 4.5 and 6. Additionally, the effect of temperature on enzyme activity (Figure S7) further revealed the temperature dependence of the nanozyme’s catalytic activity. The results showed that nanozymes with different AA modifications exhibited a “rise-then-fall” pattern of activity across a temperature range of 4 to 90 °C, with the highest relative activity typically observed between 20 and 50 °C. The condition optimization experiments indicated that weakly acidic environments (pH 4.5–6) and moderate temperature ranges (20–50 °C) provided the optimal reaction conditions for the modified nanozymes, which closely match the conditions found in many biological systems. Ultimately, pH = 5 and room temperature were selected as the best conditions for subsequent detection applications.Furthermore, the long-term stability of Cu₂O@AA was tested, and the results showed that no significant loss of enzymatic activity occurred within 100 days, indicating that AA-modified Cu₂O exhibits good long-term stability (Figure S8).

Furthermore, enzyme kinetics experiments were conducted to assess the differences in POD-like activity of Cu₂O modified with various AA. As shown in Fig. 3 A1–D1 and A2–D2, the catalytic reaction data under varying H₂O₂ concentrations were fitted to calculate the *K*_m_ and *V*_max_. All reactions exhibited a rapid increase in velocity with increasing H₂O₂ concentration, followed by a plateau, which is characteristic of typical enzymatic kinetics. The Lineweaver–Burk double-reciprocal fitting yielded high *R*^2^ values, indicating that the experimental data align well with the Michaelis–Menten kinetic model. This confirms that Cu₂O@AA nanocomposites exhibit kinetic properties similar to those of natural peroxidase enzymes. Notably, the *K*_m_ values for different nanocomposites revealed their varying affinities for H₂O₂. Among them, Cu₂O@Arg demonstrated the lowest *K*_m_, indicating its superior affinity for H₂O₂. Similarly, the catalytic velocity for TMB oxidation was also concentration-dependent (Fig. 3 A3–D3 and A4–D4). The Lineweaver–Burk double-reciprocal plot analysis showed that Cu₂O@Gly exhibited the highest *V*_max_, suggesting its superior catalytic efficiency for TMB oxidation. These results indicate that different AAs significantly affect the catalytic activity of Cu₂O, which can be attributed to the variation in substrate interactions mediated by the AAs and their structural differences [24].

In addition, a comparison with previously reported nanozymes (Table S1) demonstrated that the POD-like activity of Cu₂O@AA nanocomposites exhibits significant competitive advantages. This highlights the enhancement in catalytic performance achieved through AA modification of Cu₂O nanoparticles.

### Construction and application of colorimetric sensing arrays

**Fig. 4:**
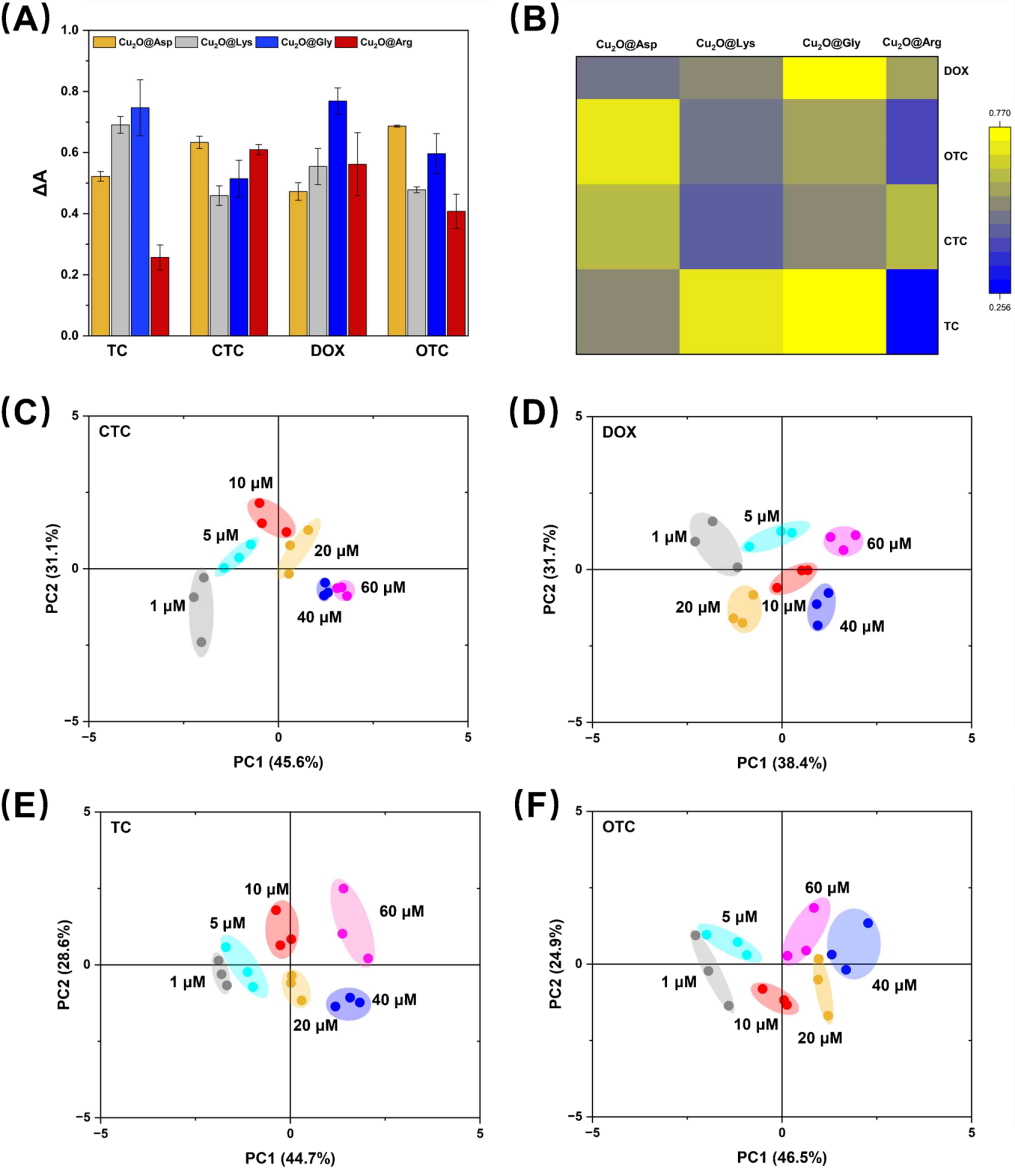
**A** Differential absorbance (Δ*A*) of the enzyme-like activities of Cu_2_O@Asp, Cu_2_O@Lys, Cu_2_O@Gly, and Cu_2_O@Arg in relation to four antibiotics: tetracycline (TC), chlortetracycline (CTC), doxycycline (DOX), and oxytetracycline (OTC) (*n* = 3). **B** Heat map showing the effect of activity between different Cu_2_O@AA nanozymes and antibiotics. Higher values indicate stronger inhibition of enzyme activity. **C**–**F** Discriminant two-dimensional score plots of four different concentrations of antibiotics (CTC, DOX, TC, OTC) in colorimetric sensing arrays. Concentrations tested included 1, 5, 10, 20, 40, and 60 µM

Based on the differentiated POD-like activities of four different nanozymes (Cu₂O@Asp, Cu₂O@Lys, Cu₂O@Gly, and Cu₂O@Arg), a colorimetric sensing array was constructed for the rapid and sensitive detection of TCs (TC, CTC, DOX, and OTC). Figure 4A shows the colorimetric responses (represented as ΔA, i.e., changes in absorbance) of the four nanozymes in the detection of different tetracycline antibiotics. The results indicate significant differences in the enzymatic activity of the four nanozymes across the various antibiotics. To further visually demonstrate the response patterns of different nanozymes to TCs, a heatmap was used in Fig. 4B to represent the differences in colorimetric response values, with color gradation from blue to yellow corresponding to lower and higher response values, respectively. The heatmap clearly reflects the varying responses of each nanozyme to the same antibiotic, providing a basis for distinguishing different antibiotics through the combination of nanozyme response patterns [33].

To verify the ability of the colorimetric sensing array to differentiate between various antibiotics and their concentrations, PCA was performed for dimensionality reduction [34–36], and the results are shown in Fig. 4C–F. Each plot corresponds to one antibiotic (CTC, DOX, TC, and OTC), with the two-dimensional coordinates representing the sample distribution in the principal component space (PC1 and PC2). Different colors of points represent different concentrations (1 to 60 μM) of TCs. Figure 4C and D show that the concentration gradients of CTC and DOX exhibit clear distribution trends: higher concentrations are increasingly separated from lower concentrations in the principal component space. For example, CTC data points at low concentrations (1 μM and 5 μM) are clustered in the lower left corner of the principal component space, while at high concentration (60 μM), they are distributed in the upper right corner. Similarly, Fig. 4E and F display the concentration distribution of TC and OTC. Compared to CTC and DOX, the data points for TC and OTC are more concentrated at lower concentrations (1 to 10 μM), but as the concentration increases, the points gradually separate, with high-concentration samples showing a distinct shift in the principal component space. This result suggests that the colorimetric sensing array has high sensitivity for detecting low-concentration antibiotics and strong accuracy in distinguishing high-concentration samples.

Furthermore, the cumulative contribution rates of the principal components PC1 and PC2 in the PCA analysis were relatively high (e.g., PC1 and PC2 contributed 44.7% and 28.6%, respectively, as shown in Fig. 4E), further demonstrating the strong classification ability and explanatory power of the response data from the sensing array. This multidimensional data analysis method fully illustrates the superiority and potential application value of the colorimetric sensing array in the analysis of complex samples.

**Fig. 5:**
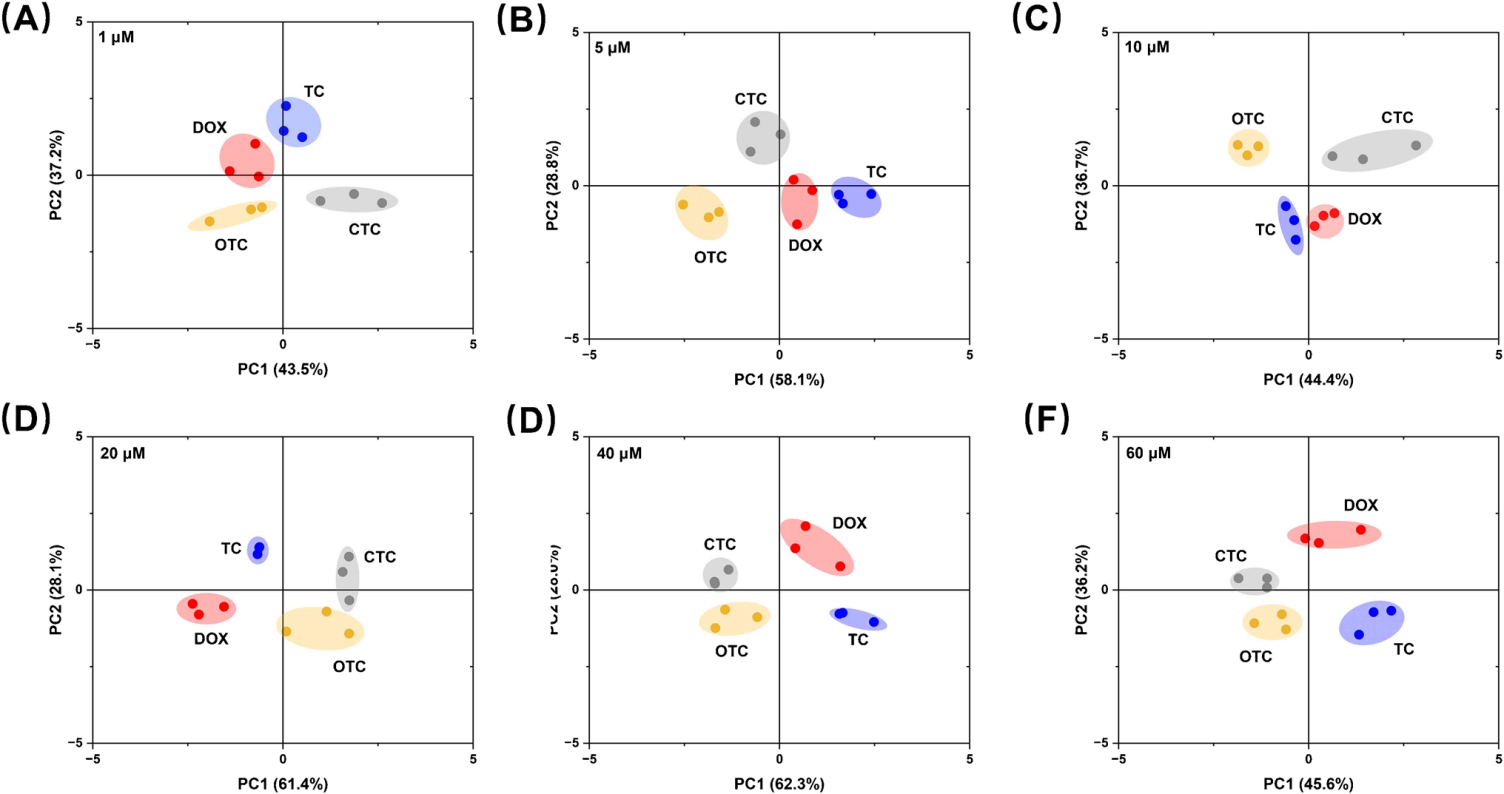
Performance of colorimetric sensing array in differentiating four antibiotics (TC, DOX, OTC, and CTC) and their concentration gradients. **A**–**F** PCA results demonstrating discriminatory two-dimensional scoring plots. The distribution of sample points at different concentration gradients (1 μM to 60 μM) was **A** 1 μM, **B** 5 μM, **C** 10 μM, **D** 20 μM, **E** 40 μM, and **F** 60 μM

To further investigate the overall performance of the colorimetric sensor array in distinguishing multiple TCs and their concentration gradients, PCA was applied to analyze the data from different antibiotics (TC, DOX, OTC, and CTC) at various concentration gradients, as shown in Fig. 5A-F. At a low concentration of 1 μM (Fig. 5A), the sample points of each antibiotic were relatively clustered in the principal component space, indicating minimal colorimetric response differences among the four antibiotics at low concentrations. However, as the concentration increased (Fig. 5B-F), the sample points of different antibiotics gradually exhibited distinct distribution patterns. For instance, starting from Fig. 5B, the sample points of DOX progressively deviated from the center along the PC1 dimension as the concentration increased, whereas the sample points of TC showed more pronounced shifts along the PC2 dimension (Fig. 5C, E). The concentration gradient distribution trends of OTC and CTC also demonstrated significant changes in the principal component space, particularly at higher concentrations (40 μM and 60 μM, Fig. 5E and F), where clear classification regions were formed for each antibiotic.

Moreover, the cumulative contribution rates of PC1 and PC2 further reflect the enhanced classification capability of PCA analysis as the antibiotic concentration increases. For example, in Fig. 5F, the cumulative contribution rate of PC1 and PC2 reached 81.8%, which was significantly higher than the 71.7% observed in Fig. 5A. These findings further validate the potential of the colorimetric sensor array combined with multidimensional data analysis methods for the classification and identification of complex samples. Compared to the previous single-target detection methods using copper-based nanozymes [37, 38], this colorimetric sensing array system can simultaneously identify four classes of antibiotics, significantly enhancing detection efficiency through its multi-target detection capability. A systematic evaluation was conducted to assess the performance of the colorimetric sensor array in distinguishing binary antibiotic mixtures, its anti-interference capability, and its applicability to municipal wastewater samples, providing a scientific basis for the efficient analysis and identification of complex samples.

In the detection of binary antibiotic mixtures, OTC, TC, DOX, and CTC were selected as target analytes. PCA models were constructed based on the response data generated by the colorimetric sensor array. The results demonstrated that different antibiotic combinations (e.g., OTC + TC, OTC + DOX, CTC + TC) exhibited clear separation in the principal component space, with minimal overlap between sample distributions in the PC1 and PC2 dimensions. This outcome highlights the high resolution of the colorimetric sensor array in detecting multiple targets. Notably, the cumulative contribution of PC1 and PC2 reached 65.7%, underscoring the efficacy of PCA in capturing response differences between binary mixtures.

To verify anti-interference capability, a detection system was designed wherein target antibiotics coexisted with interfering substances, including common impurity ions found in aqueous environments (5 mM:Na⁺, K⁺, Ca2⁺, Mg2⁺, Cu2⁺, Cl⁻, SO₄2⁻, NO₃⁻, HCO₃⁻) and organic molecules (2 mM: glucose, penicillin, streptomycin, lysine, glutamic acid). PCA analysis revealed that the sample points of interfering substances were significantly separated from those of the target antibiotics in the principal component space. The sample points for each antibiotic maintained stable distributions and robust classification characteristics. These results demonstrate that the colorimetric sensor array exhibits high selectivity for target molecules in complex systems and demonstrates strong robustness and reliability against interference.

**Fig. 6:**
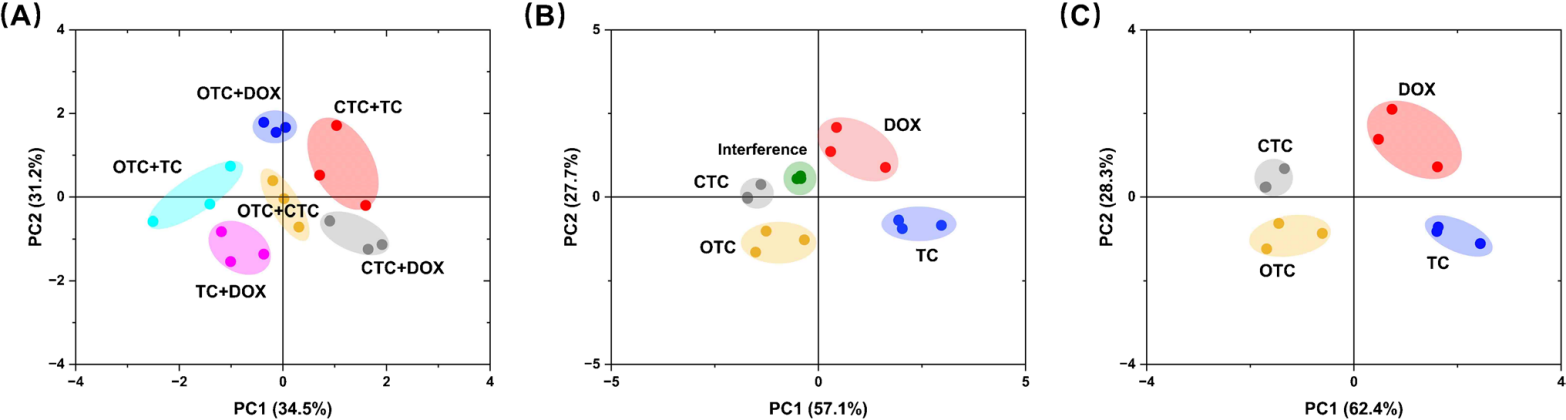
**A** Discriminant score plots for binary antibiotic mixtures (OTC + TC, OTC + DOX, CTC + TC, OTC + CTC, TC + DOX, and CTC + DOX). **B** Discriminant score plot of potential interfering substances with TCs. **C** Discriminant score plot of four TCs in municipal wastewater

In the detection of municipal wastewater samples, the environmental adaptability and practical application potential of the colorimetric sensor array were further validated. After simple pretreatment, municipal wastewater samples spiked with different antibiotics were analyzed using the colorimetric sensor array. PCA results showed that the sample points of different antibiotics in the municipal wastewater matrix displayed distinct separation trends (Fig. 6C), and the distribution characteristics of the concentration gradient samples in the principal component space were highly consistent with the data obtained under laboratory conditions. Particularly, when the cumulative contribution of PC1 and PC2 exceeded 90%, the sensor array demonstrated excellent classification performance and environmental tolerance.

In conclusion, this study systematically evaluated the performance of the colorimetric sensor array, comprehensively revealing its significant potential for classification in complex samples, anti-interference detection, and practical environmental applications.

## Conclusion

In this study, we successfully synthesized four copper-based nanozymes, Cu₂O@Asp, Cu₂O@Arg, Cu₂O@Gly, and Cu₂O@Lys, by utilizing different AA to regulate their catalytic activity. The results show that the type of AA significantly influences the particle size, morphology, and POD-like catalytic activity of the nanoparticles. This finding effectively demonstrates the potential of small molecules to regulate the properties of nanozymes and provides a universal and efficient strategy for optimizing the catalytic performance of nanomaterials. Furthermore, based on these four AA-regulated copper-based nanozymes, we successfully constructed a highly sensitive, easy-to-operate, and cost-effective colorimetric sensor array. This sensor array can effectively distinguish and detect common TCs, showing broad application prospects in environmental monitoring and food safety. In conclusion, this study not only provides important theoretical support for the small-molecule regulation of copper-based nanozyme activity but also offers a novel approach for the detection of TCs.

## Supplementary Information

Below is the link to the electronic supplementary material.

Supplementary file1 (DOCX 2229 KB)

## Data Availability

Supporting data can be obtained from the corresponding author upon reasonable request.

## References

[1] Zhang R, Yan X, Fan K. Nanozymes inspired by natural enzymes. Acc Mater Res. 2021;2(7):534–47. 10.1021/accountsmr.1c00074.

[2] Wang Q, Wei H, Zhang Z, Wang E, Dong S. Nanozyme: an emerging alternative to natural enzyme for biosensing and immunoassay. TrAC Trends Anal Chem. 2018;105:218–24. 10.1016/j.trac.2018.05.012.

[3] Li J, Wu Y, Qin Y, Liu M, Chen G, Hu L, Gu W, Zhu C. AgCu@CuO aerogels with peroxidase-like activities and photoelectric responses for sensitive biosensing. Chem Commun. 2021;57(100):13788–91. 10.1039/d1cc06177a. 10.1039/d1cc06177a 34870654

[4] Fu R, Ma Z, Zhao H, Jin H, Tang Y, He T, Ding Y, Zhang J, Ye D. Research progress in iron-based nanozymes: catalytic mechanisms, classification, and biomedical applications. Anal Chem. 2023;95(29):10844–58. 10.1021/acs.analchem.3c01005. 37438259 10.1021/acs.analchem.3c01005

[5] Zhang Y, Ni S, Chong C, Xu J, Mu X, Zhang X-D. Biocatalysts at atom level: from coordination structure to medical applications. Appl Mater Today. 2021;23. 10.1016/j.apmt.2021.101029.

[6] Meng L, Tang L, Gao F, Zhu L, Liu X, Zhang J, Chang Y, Ma X, Guo Y. Hollow CeO2-based nanozyme with self-accelerated cascade reactions for combined tumor therapy. Chem A Eur J. 2024;30(49). 10.1002/chem.202401640. 10.1002/chem.202401640 38935332

[7] Diao Q, Chen X, Tang Z, Li S, Tian Q, Bu Z, Liu H, Liu J, Niu X. Nanozymes: powerful catalytic materials for environmental pollutant detection and degradation. Environ Sci Nano. 2024;11(3):766–96. 10.1039/d3en00844d.

[8] Li S, Chen Z, Yang F, Yue W. The age of vanadium-based nanozymes: synthesis, catalytic mechanisms, regulation and biomedical applications. Chin Chem Lett. 2024;35(4). 10.1016/j.cclet.2023.108793.

[9] Ma Q, Liu Y, Zhu H, Zhang L, Liao X. Nanozymes in tumor theranostics. Front Oncol. 2021;11. 10.3389/fonc.2021.666017. 10.3389/fonc.2021.666017 PMC8560966 34737942

[10] Hou J, Xianyu Y. Tailoring the surface and composition of nanozymes for enhanced bacterial binding and antibacterial activity. Small. 2023;19(42). 10.1002/smll.202302640. 10.1002/smll.202302640 37322391

[11] Ding S, Barr JA, Lyu Z, Zhang F, Wang M, Tieu P, Li X, Engelhard MH, Feng Z, Beckman SP, Pan X, Li J-C, Du D, Lin Y. Effect of phosphorus modulation in iron single-atom catalysts for peroxidase mimicking. Adv Mater. 2024;36(10). 10.1002/adma.202209633. 10.1002/adma.202209633 36722360

[12] Zhang D, Chen Q, Ren Q, Zhong W, Zhang H, Wang G, Zhang Y. Transition metal-based nanozymes: classification, catalytic mechanisms and emerging biomedical applications. Coord Chem Rev. 2024;508. 10.1016/j.ccr.2024.215771.

[13] Gao Y, Zhu Z, Chen Z, Guo M, Zhang Y, Wang L, Zhu Z. Machine learning in nanozymes: from design to application. Biomater Sci. 2024;12(9):2229–43. 10.1039/d4bm00169a. 38497247 10.1039/d4bm00169a

[14] Liu B, Liu J. Surface modification of nanozymes. Nano Res. 2017;10(4):1125–48. 10.1007/s12274-017-1426-5.

[15] Chen Z, Li S, Yang F, Yue W. Construction of a colorimetric sensor array for the identification of phenolic compounds by the laccase-like activity of N-doped manganese oxide. Talanta. 2024;268. 10.1016/j.talanta.2023.125324. 10.1016/j.talanta.2023.125324 37951179

[16] Karthiga D, Choudhury S, Chandrasekaran N, Mukherjee A. Effect of surface charge on peroxidase mimetic activity of gold nanorods (GNRs). Mater Chem Phys. 2019;227:242–9. 10.1016/j.matchemphys.2019.02.015.

[17] Li S, Chen Z, Yang F, Qiao C, Zhang S, Yue W. Highly active oxidase-like N-Mn3O4 nanoparticles from amino acid nitrogen source for dual-mode detection of acid phosphatase. ACS Appl Nano Mater. 2023. 10.1021/acsanm.3c02243.37649833

[18] Shome A, Ali S, Haydar MS, Sarkar K, Roy S, Adhikary P, Roy MN. Synthesis of spherical Mn2O3 nanozymes from different green precursors for their innovative applications in catalytic properties and bioactivity. ACS Biomater Sci Eng. 2024;10(3):1734–42. 10.1021/acsbiomaterials.3c00608. 38330433 10.1021/acsbiomaterials.3c00608

[19] Li C, Hang T, Jin Y. Atomically Fe-anchored MOF-on-MOF nanozyme with differential signal amplification for ultrasensitive cathodic electrochemiluminescence immunoassay. Exploration. 2023;3(4). 10.1002/exp.20220151. 10.1002/EXP.20220151 PMC10624370 37933237

[20] Li X, Ding S, Lyu Z, Tieu P, Wang M, Feng Z, Pan X, Zhou Y, Niu X, Du D, Zhu W, Lin Y. Single-atomic iron doped carbon dots with both photoluminescence and oxidase-like activity. Small. 2022;18(37). 10.1002/smll.202203001. 10.1002/smll.202203001 35986440

[21] Zhan L, Xia Z, Xu Z, Xie B. Study on the remediation of tetracycline antibiotics and roxarsone contaminated soil. Environ Pollut. 2021;271. 10.1016/j.envpol.2020.116312. 10.1016/j.envpol.2020.116312 33360583

[22] Wang C-Y, Wang C-C, Zhang X-W, Ren X-Y, Yu B, Wang P, Zhao Z-X, Fu H. A new Eu-MOF for ratiometrically fluorescent detection toward quinolone antibiotics and selective detection toward tetracycline antibiotics. Chin Chem Lett. 2022;33(3):1353–7. 10.1016/j.cclet.2021.08.095.

[23] Rong M, Huang Y, Lin C, Lai L, Wu Y, Niu L. Recent advances in optical sensing for tetracycline antibiotics. TrAC Trends Anal Chem. 2024;178. 10.1016/j.trac.2024.117839.

[24] Li S, Chen Z, Liu M, Yang F, Zhang S, Qiao C, Zhong W, Yue W. Ultrasmall Cu2O@His with laccase- and catechol oxidase-like activity: applications in phenolic drug identification and degradation. Chem Eng J. 2024;485. 10.1016/j.cej.2024.150058.

[25] Li S, Chen Z, Wang M, Yang F, Zhang S, Qiao C, Chu W, Yue W. Ultrasmall Cu2O@His nanozymes with RONS scavenging capability for anti-inflammatory therapy. ACS Appl Mater Interfaces. 2024;16(3):3116–25. 10.1021/acsami.3c15083. 38224533 10.1021/acsami.3c15083

[26] Zheng J-J, Zhu F, Song N, Deng F, Chen Q, Chen C, He J, Gao X, Liang M. Optimizing the standardized assays for determining the catalytic activity and kinetics of peroxidase-like nanozymes. Nat Protoc. 2024;19(12):3470–88. 10.1038/s41596-024-01034-7. 39147983 10.1038/s41596-024-01034-7

[27] Hao C, Qu A, Xu L, Sun M, Zhang H, Xu C, Kuang H. Chiral molecule-mediated porous CuxO nanoparticle clusters with antioxidation activity for ameliorating Parkinson’s disease. J Am Chem Soc. 2019;141(2):1091–9. 10.1021/jacs.8b11856. 30540450 10.1021/jacs.8b11856

[28] Wu D, Liu P, Wang T, Chen X, Yang L, Jia D. Amino acid-assisted synthesis of Fe2O3nitrogen doped graphene hydrogels as high performance electrode material. Electrochim Acta. 2018;283:1858–70. 10.1016/j.electacta.2018.07.103.

[29] Chen Z, Li S, Guan Y, Wu C, Qian Y, Zhou H, Qian Y, Yue Y, Yue W. Ultrasmall CuMn-His nanozymes with multienzyme activity at neutral ph: construction of a colorimetric sensing array for biothiol detection and disease identification. ACS Appl Mater Interfaces. 2024. 10.1021/acsami.4c04844. 38940445 10.1021/acsami.4c04844

[30] Jiang L, Yao M, Liu B, Li Q, Liu R, Lv H, Lu S, Gong C, Zou B, Cui T, Liu B, Hu G, Wagberg T. Controlled synthesis of CeO2/graphene nanocomposites with highly enhanced optical and catalytic properties. J Phys Chem C. 2012;116(21):11741–5. 10.1021/jp3015113.

[31] Li S, Chen Z, Yang F, Yue W. Self-template sacrifice and in situ oxidation of a constructed hollow MnO2 nanozymes for smartphone-assisted colorimetric detection of liver function biomarkers. Anal Chim Acta. 2023;1278. 10.1016/j.aca.2023.341744. 10.1016/j.aca.2023.341744 37709473

[32] Yang J, Xiao S, Deng J, Li Y, Hu H, Wang J, Lu C, Li G, Zheng L, Wei Q, Zhong J. Oxygen vacancy-engineered cerium oxide mediated by copper-platinum exhibit enhanced SOD/CAT-mimicking activities to regulate the microenvironment for osteoarthritis therapy. J Nanobiotechnol. 2024;22(1). 10.1186/s12951-024-02678-z. 10.1186/s12951-024-02678-z PMC11330606 39155382

[33] Xi H, Gu H, Han Y, You T, Wu P, Liu Q, Zheng L, Liu S, Fu Q, Chen W, Gao Y, Wang Y, Yin P. Peroxidase-like single Fe atoms anchored on Ti3C2Tix MXene as surface enhanced Raman scattering substrate for the simultaneous discrimination of multiple antioxidants. Nano Res. 2023;16(7):10053–60. 10.1007/s12274-023-5739-2.

[34] Sharifi H, Tashkhourian J, Hemmateenejad B. An array of metallic nanozymes can discriminate and detect a large number of anions. Sensors Actuators B Chem. 2021;339. 10.1016/j.snb.2021.129911.

[35] Li F, Jiang J, Peng H, Li C, Li B, He J. Platinum nanozyme catalyzed multichannel colorimetric sensor array for identification and detection of pesticides. Sensors Actuators B Chem. 2022;369. 10.1016/j.snb.2022.132334.

[36] Zhou X, Li L, Wang Y, Kong T, Cao Z, Xie H, Liang W, Wang Y, Qian S, Chao J, Zheng J. Nanozyme inhibited sensor array for biothiol detection and disease discrimination based on metal ion-doped carbon dots. Anal Chem. 2023;95(23):8906–13. 10.1021/acs.analchem.3c00601. 37265323 10.1021/acs.analchem.3c00601

[37] Ai W, Chen G, Chen J, Jin Y, Wang X, Zhou T, Zhang Z, Wang F, Zhang G. Cu-MoOx-based nanozyme with enhanced peroxidase like activity for quinolone antibiotics detection. Spectrochim Acta A Mol Biomol Spectrosc. 2025;325. 10.1016/j.saa.2024.125117. 10.1016/j.saa.2024.125117 39288602

[38] Liu M, Wang Y, Xiao J, Liu Y, Ren Y, Gao X. Colorimetric immunoassay of furazolidone metabolites based on iron-copper bimetallic organic framework nanoenzyme. Microchim Acta. 2024;191(10). 10.1007/s00604-024-06657-x. 10.1007/s00604-024-06657-x 39235626

