# Supplementary material for: Amino acid regulation of peroxidase-like activity of Cu2O nanozyme for detection of tetracycline antibiotics
Source: Anal Bioanal Chem. 2025 May 14;417(17):3813–23. doi: 10.1007/s00216-025-05904-1 (PMC12227342; doi:10.1007/s00216-025-05904-1)
Supplement: Supplementary file 1 — Supplementary file1 (DOCX 2229 KB) [file 216_2025_5904_MOESM1_ESM.docx]

**SUPPORTING INFORMATION**

**Amino Acid Regulation of Peroxidase-like Activity of Cu_2_O Nanozyme for Detection of Tetracycline Antibiotics**

Yueqiang wang ^a^, Shengwei Sun^,b^^[[1]](#footnote-1)^*

^a^ Faculty of Science, National University of Singapore, Block S16 Level 9, 6 Science Drive 2 Singapore 117546

^b^School of Engineering Sciences in Chemistry, Biotechnology and Health, Department of Fibre and Polymer Technology, KTH Royal Institute of Technology, 100 44, Stockholm, Sweden

| 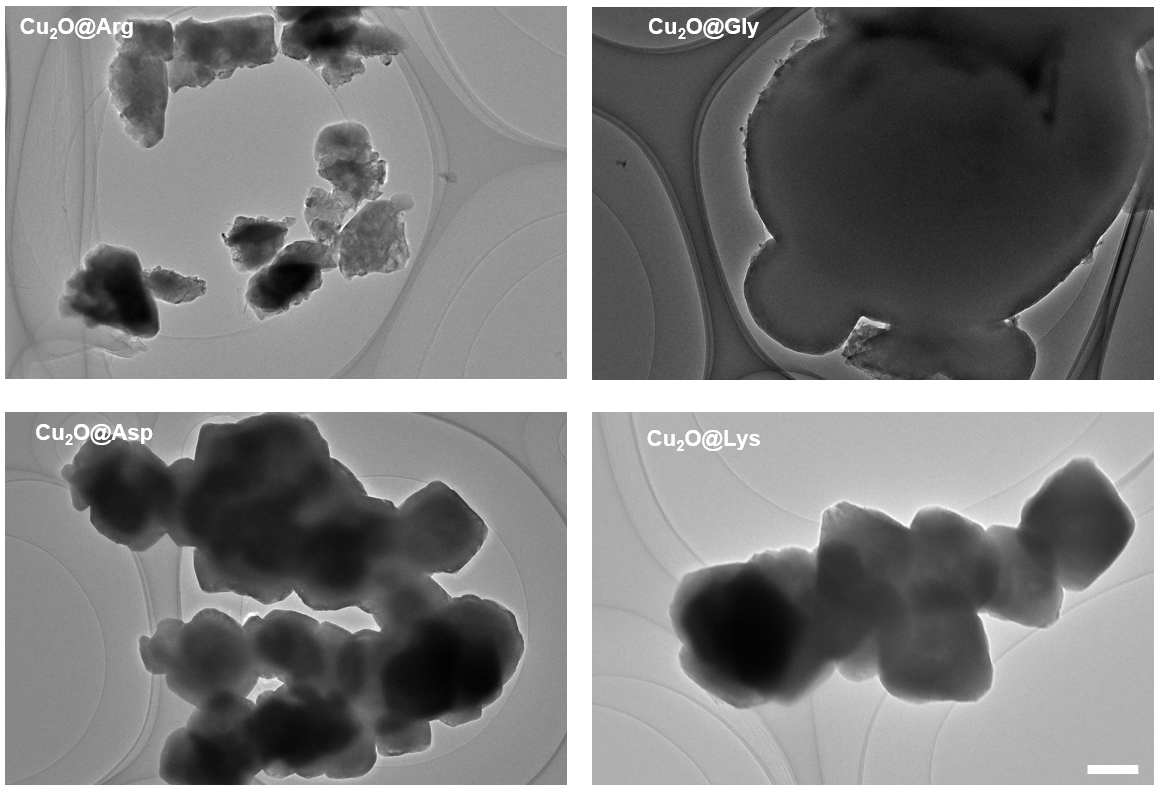 |
| --- |
| **Figure S1.** Transmission electron microscopy images of Cu₂O particles with different amino acid modifications. Scale bars, 200 nm. |
|  |

|  |
| --- |
| Figure S2.Hydrated particle size of Cu₂O with different amino acid modifications, Hydrated particle size of Cu₂O with different amino acid modifications. |

|  |
| --- |
| Figure S3. Thermogravimetric analysis (TGA) of Cu₂O modified with different amino acids, (A) Cu₂O@Arg, (B) Cu₂O@Gly, Cu₂O@Asp, and Cu₂O@Lys. |

|  |
| --- |
| **Figure S4.** N_2_ adsorption-desorption isotherms of (A) Cu₂O@Arg, (B) Cu₂O@Gly, Cu₂O@Asp, and Cu₂O@Lys. The red line shows the adsorption branch and the black line shows the desorption branch.The unclosed curves may be attributed to the smaller size of the nanoenzymes not applicable with the Brunauer–Emmett–Teller model. |

|  |
| --- |
| **Figure S5.** Differences in POD-like activities of Cu_2_O modified with different amino acids and unmodified Cu_2_O. |

| 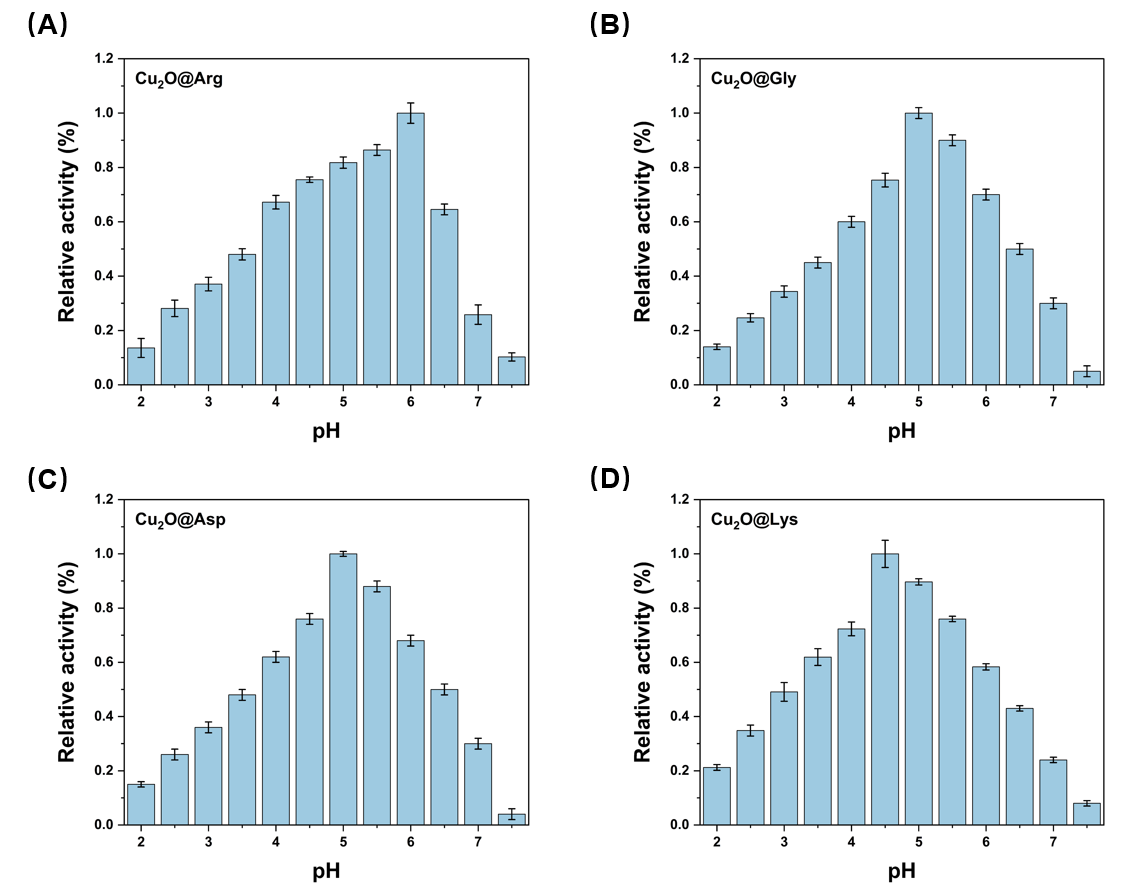 |
| --- |
| **Figure S6.** Relative POD-like activities of Cu₂O particles modified with different amino acids at different pH. |

| 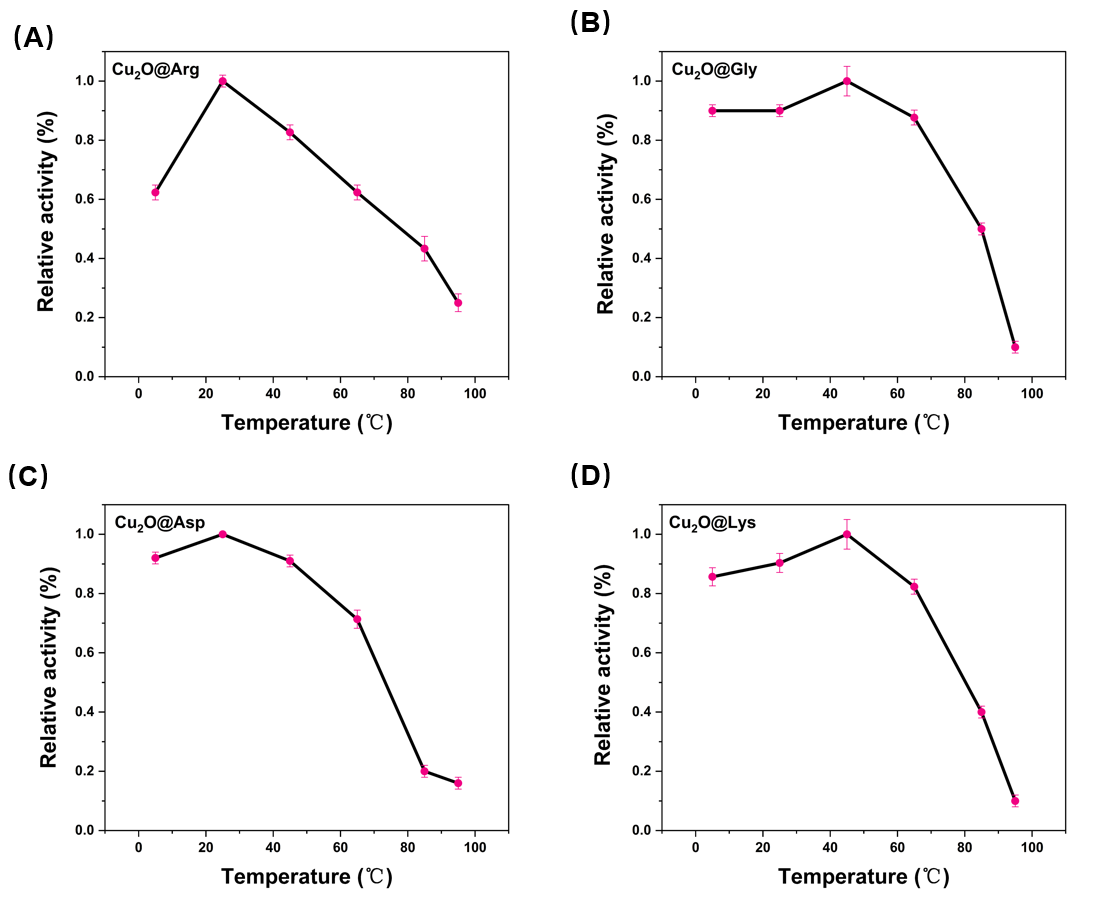 |
| --- |
| **Figure S7.** Relative POD-like activities of Cu₂O particles with different amino acid modifications at different temperatures. |
|  |
|  |





**Figure S8.** Relative POD-like activity of the same batch of Cu₂O@AA particles at different times.

|  | Table S1. Comparison of the kinetic parameters of laccase-like nanomaterials and natural laccase toward typical substrates. | | | | | |
| --- | --- | --- | --- | --- | --- | --- |
| **Catalyst** | | **H_2_O_2_** | | **TMB** | | **Ref.** |
|  | | ***K*^m^(mM)** | ***V^max^* (10^-8^ M s^−1^)** | ***K*^m^(mM)** | ***V^max^* (10^-8^M s^−1^)** |  |
| HRP | | 3.7 | 8.71 | 0.434 | 10 | ^1^ |
| Cu-MOF | | 28.58 | 5.45 | 0.456 | 2.478 | ^2^ |
| Cu–Ag/rGO | | 8.62 | 7.01 | 4.25 | 0.011 | ^3^ |
| CuNPs-N/C | | 17.98 | 12.8 | 1.57 | 8.57 | ^4^ |
| Fe–N–C SAzyme | | 12.2 | 35.6 | 3.6 | 116 | ^5^ |
| Cu-curcumin | | 7.92 | 14.5 | 0.127 | 5.51 | ^6^ |
| **Cu_2_O@Asp** | | 22.54 | 40.98 | 0.070 | 16.81 | **This work** |
| **Cu_2_O@Gly** | | 19.7 | 36.63 | 0.110 | 18.62 |  |
| **Cu_2_O@Lys** | | 17.7 | 33.0 | 0.050 | 17.21 |  |
| **Cu_2_O@Arg** | | 6.79 | 15.47 | 0.080 | 12.77 |  |

# References

1. Y. Zhang, Z. Zhou, F. Wen, J. Tan, T. Peng, B. Luo, H. Wang and S. Yin, *Sens. Actuators, B*, 2018, **275**, 155-162.

2. H. Yu, H. Wu, X. Tian, Y. Zhou, C. Ren and Z. Wang, *RSC Adv.*, 2021, **11**, 26963-26973.

3. G. Darabdhara, B. Sharma, M. R. Das, R. Boukherroub and S. Szunerits, *Sens. Actuators, B*, 2017, **238**, 842-851.

4. Y. Wu, J. Wu, L. Jiao, W. Xu, H. Wang, X. Wei, W. Gu, G. Ren, N. Zhang and Q. Zhang, *Anal. Chem.*, 2020, **92**, 3373-3379.

5. L. Jiao, W. Xu, H. Yan, Y. Wu, C. Liu, D. Du, Y. Lin and C. Zhu, *Anal. Chem.*, 2019, **91**, 11994-11999.

6. Z. Chai, A. Zhou, J. Huang, L. Qu, J. Ge, L. Zhang and Z. Li, *The Analyst*.

1. * Corresponding author.

   E-mail addresses: shengw@kth.se. [↑](#footnote-ref-1)
